# Drought and High Temperatures Impact the Plant–Pollinator Interactions in *Fagopyrum esculentum*

**DOI:** 10.3390/plants14010131

**Published:** 2025-01-04

**Authors:** Corentin Defalque, Joy Laeremans, Jonathan Drugmand, Chanceline Fopessi Tcheutchoua, Yu Meng, Meiliang Zhou, Kaixuan Zhang, Muriel Quinet

**Affiliations:** 1 Earth and Life Institute-Agronomy, Université Catholique de Louvain, B-1348 Louvain-la-Neuve, Belgium; corentin.defalque@uclouvain.be (C.D.); my131sohu@126.com (Y.M.); 2 Country College of Landscape Architecture and Tourism, Hebei Agricultural University, Baoding 071001, China; 3 State Key Laboratory of Crop Gene Resources and Breeding, Institute of Crop Sciences, Chinese Academy of Agricultural Sciences, Beijing 100081, China; zhoumeiliang@caas.cn (M.Z.); zhangkaixuan@caas.cn (K.Z.)

**Keywords:** buckwheat, pollination, drought, high temperatures, stress, plant–pollinator interactions, *F. esculentum*

## Abstract

As a result of climate change, temperate regions are facing the simultaneous increase in water and heat stress. These changes may affect the interactions between plants and pollinators, which will have an impact on entomophilous crop yields. Here, we investigated the consequences of high temperatures and water stress on plant growth, floral biology, flower-reward production, and insect visitation of five varieties of common buckwheat (*Fagopyrum esculentum*), an entomophilous crop of growing interest for sustainable agriculture. The plants were grown under two temperature regimes (21 °C/19 °C and 28 °C/26 °C, day/night) and two watering regimes (well-watered and water-stressed). Our results showed that the reproductive growth was more affected by drought and high temperatures than was the vegetative growth, and that combined stress had more detrimental effects. However, the impact of drought and high temperatures was variety-dependent. Drought and/or high temperatures reduced the number of open flowers per plant, as well as the floral resources (nectar and pollen), resulting in a decrease in pollinator visits, mainly under combined stress. Although the proportion of Hymenoptera visiting the flowers decreased with high temperatures, the proportion of Diptera remained stable. The insect visiting behavior was not strongly affected by drought and high temperatures. In conclusion, the modification of floral display and floral resources induced by abiotic stresses related to climate change alters plant–pollinator interactions in common buckwheat.

## 1. Introduction

Climate change causes temperatures to rise. The average global temperature is expected to increase by 2.5 to 4.5 °C by the end of the century [1,2]. For temperate regions, thermic and water stress will occur more often and simultaneously during the spring and summer seasons [3]. These periods constitute crucial moments for plant–pollinator interactions, as these organisms are strongly interdependent. Nectar is the primary source of sugar and energy for flying insects, and the yield of entomophilous crops can be improved up to 75% with an optimal pollination [4,5]. Monitoring drought and heat stress is therefore crucial, as it can lead to a phenological decoupling of the mutualistic relationship between plants and insects [6]. Drought and high temperature must be considered together, as their impacts exhibit synergistic effects that surpass the cumulative impact of each isolated factor [7].

Concerning the development of plants, each individual stress is well characterized, but the combination is still poorly studied [8]. Globally, the combination decreases the life cycle, affects carbon assimilation and the nitrogen cycle, and promotes lipid peroxidation [9,10]. In plant tissues, heat and drought have adverse effects on essential physiological processes, including stomatal conductance, photosynthesis, and respiration [11]. Furthermore, conflicting responses may occur. During high temperature episodes, leaf stomata open to cool the plant through transpiration, whereas water conservation may take precedence during periods of drought [10].

The reproductive stage is particularly vulnerable to the detrimental effects of high temperature and drought. For female organs, the stigmata can decrease in size with a reduction of the turgor of the papilla cells and a malformation of the cortical and transmitting cells [12,13,14]. Ovary or seed abortion is a common occurrence during these stress periods [9,15,16,17]. Within male organs, pollen shedding and anther dehiscence occurs more frequently [18,19,20]. The grain stage is also affected by stresses, with a shorter filling period and reduced seed production [9,15,16,17]. In general, the reproductive stages exhibit reduced fertility [12,13].

Regarding pollinators, the abiotic factors of drought, high temperature, and their combination significantly impact their physiology and their interactions with plants. Most pollinators are ectotherms. Temperature has a direct influence on their physiology and their range of performance [21,22]. The high-temperature tolerance time is species-dependent [23]. The smaller the insect, the greater the metabolic activity will be during high-temperature events [21]. The tolerance depends also on the stage of the insect. Adults tend to be able to survive high temperatures but display reduced reproductive capacities [23].

Even if the effect of temperature is well characterized for pollinators, drought events remain under-studied for most of these, even for the honey bees *Apis mellifera* L. [24]. Social insects regulate their water resources at the colony level, while solitary insects do it individually [4]. Depending on the water source, drought will not evenly impact the different pollinator species. For instance, *Apis mellifera* can drink at the water point, whereas *Bombus* spp. depend on the water obtained from nectar [24].

Since the combination of both stressors is negatively impacting the quality of the nectar and the pollen, the behavioral patterns of insects, reliant on these primary sources of energy, will be detrimentally influenced. In the short term, a decline in quality has a negative effect on the mobility and the immunity of the pollinators [25,26]. In the long term, the lack of production and quality causes a population decline, which can be even more important when floral resources are absent, and the insect has no stocks [25,26,27,28,29,30,31].

Recently, the cultivation of pollinators-dependent crops has been increasing globally [32]. A total of 87 of 115 of the most cultivated crops depend on biotic pollination factors [33]. The annual increase in agricultural yield generated by insect accounts for 9.5% worldwide [33,34]. To maintain the productivity of those plants under climate change, heat- or drought-tolerant crops and climate-smart planting schemes are becoming essential to sustain agricultural yields [35].

Among the different existing entomophilous cultures, buckwheat (*Fagopyrum esculentum* Moench) has regained interest towards sustainable agriculture models under changing climatic conditions. This multipurpose crop is underutilized but remains important for food security in East Asia, East Europe, and the Himalayan region [36]. In recent years, this pseudocereal has regained interest for its superfood properties and its potential as a functional food ingredient. It displays a high content of dietary fibers, flavonoids, and protein, with an amino acid score of 100 [37,38,39].

Regarding its reproductive morphology, buckwheat inflorescences are compound racemes, each producing 1 to 30 uniparous cymes [40]. *Fagopyrum esculentum* is distylous, with each plant producing either pin (long-styled) flowers, or thrum (short-styled) flowers. Anthesis lasts one day, and there is only one ovule in each flower [41]. Pollen viability is high, but female sterility events are frequent [42]. These peculiarities make the seed yield extremely dependent on the capacity of the plant to attract pollinators, since the plant is self-incompatible [43].

Buckwheat relies on nectar as the key element among the different structures used by plants to facilitate pollination by insects. The attractiveness of buckwheat is linked to its capacity to produce high-quality nectar that is primarily composed of hexoses (including glucose and fructose) and sucrose, as well as vitamins and amino acids [43]. The production of buckwheat nectar also fluctuates with the age of the plant, the position in the raceme, and the morphology of the flower. Pin flowers are less visited and contain less nectar than do thrum flowers [44,45,46].

In terms of pollination, wind plays a minor role in the buckwheat’s reproductive process, whereas insect pollination significantly contributes to most of the yield [47]. The quality of the pollination may undergo significant changes according to the genetic background, the nectar productivity, the blooming stages of the plant, and the weather conditions [42]. In Europe, *Apis mellifera* and *Apis cerana* serve as the main pollinators for buckwheat; other secondary pollinators, including, *Andrena* spp., *Bombus* spp., *Osmia* spp., and various Diptera species, also contribute significantly to the overall pollination process [48,49]. The pollination guild can change with the geographic region. In Japan, the bumblebees *Bombus ardens sakagamii* and *B. hypocrite sapporoensi* are the dominant pollinating species [50]. With the upcoming environmental modifications, the reproductive physiology of buckwheat and its plant–pollinator interactions are predicted to undergo significant changes.

Drought stress has a negative impact on buckwheat floral traits and resources. Drought-stressed buckwheat manifested an increased number of flowers, concomitant with elevated rates of floral abortions and a diminished pollen viability [47]. Nectaries produce a low-quality nectar characterized by a reduced percentage of sucrose [51,52]. For pollinators, overall visitation rates of the plant are expected decrease in response to the lower quality nectar. Consequently, the diminished plant attractiveness for insects will result in reductions in both seed set and total seed mass per plant [52].

The optimum temperature range for buckwheat is between 15 °C and 25 °C. Under elevated temperature conditions, the reproductive organs will experience proportional alteration determined by both the intensity and duration of heat stress. Kreft reported that temperatures above 30 °C are deleterious, but this impact depends on the genotype or variety [53,54]. The overall consequences include an elongated flowering period and less assimilates distributed across the flowers and the seeds [55]. For the female component, the embryo sac is more prone to developmental complications, increasing the probability of degeneration and abortion [53,55,56]. The male part is less affected, since the pollen production increases, and the viability is not impacted by high temperature [57]. For the pollinators, warmer temperatures can be detrimental, depending on the insect size. Beetles, butterflies, and wasps are more active next to buckwheat under high temperatures, whereas ants and non-syrphid flies are less observed [58]. Moreover, high temperature events can modify the buckwheat–pollinator interaction by diminishing the number of floral scent compounds detected by the insects, thereby altering pollination behavior [59].

While heat- and drought-stress are frequently investigated individually in the current literature, the exploration of their combined effects on reproduction and plant–pollinator interaction remains uncharted. In this study, we assessed the response of five *Fagopyrum esculentum* varieties to high temperatures, drought stress, and their combined effects. We analyzed their reproductive physiology and observed the impact of each stress and their combination on plant–pollinator interactions.

## 2. Results

### 2.1. Impact of Drought and High Temperature on Buckwheat Growth and Flowering

Five varieties of *F. esculentum* (Table S1) were cultivated in a greenhouse, with each subjected to two temperature and watering regimes. The plants were grown under two temperature conditions: 21 °C/19 °C (day/night) under either well-watered (21 °C WW) or water-stressed (21 °C WS) conditions, and 28 °C/26 °C (day/night), under either well-watered (28 °C WW) or water-stressed (28 °C WS) conditions (Figure S1). Experiments were performed between 2018 and 2023. All the plants remained alive throughout the experiment, regardless of the treatment.

#### 2.1.1. Leaf Production

**Figure 1:**
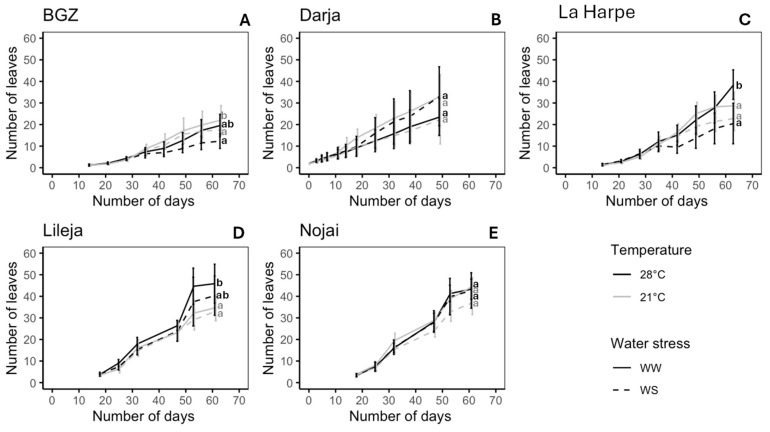
Leaf production per plant of *F. esculentum* varieties subjected to 21 °C vs. 28 °C and well-watered (WW) vs. water-stressed (WS) conditions. Varieties: (**A**) Brabantse grijze zandboekweit (BGZ), (**B**) Darja, (**C**) La Harpe, (**D**) Lileja, and (**E**) Nojai. Data are means ± sd. Values followed by the same letter for the same variety were not statistically significant at the 5% level at the end of the experiment.

The effect of high temperature and drought on leaf production varied according to the variety (Figure 1, Table S2). In Nojai and Darja, the number of leaves at the end of the experiment was not significantly affected by heat and drought, although a significant interaction between these factors was observed for Darja (Figure 1B,E, Table S2). Brabantse grijze zandboekweit (BGZ) was the only variety showing a decrease in leaf production when exposed to both heat and drought (Table S2), so that at the end of the experiment, the number of leaves was 44% lower at 28 °C WS compared to the number at 21 °C WW (Figure 1A). In La Harpe, leaf production was significantly reduced by water stress, especially at 28 °C, while in Lileja, leaf production was significantly affected by high temperatures (Figure 1C,D, Table S2). However, for both varieties, the highest leaf production was observed at 28 °C WW.

#### 2.1.2. Inflorescence Production

Depending on the variety, the first inflorescence emerged between the 4th and the 8th node (Table 1). High temperature and drought did not affect the flowering time, except in La Harpe (Table 1 and Table S2). For this variety, the first inflorescence gradually appeared later under the control, single-stress, or double-stress conditions.

**Figure 2:**
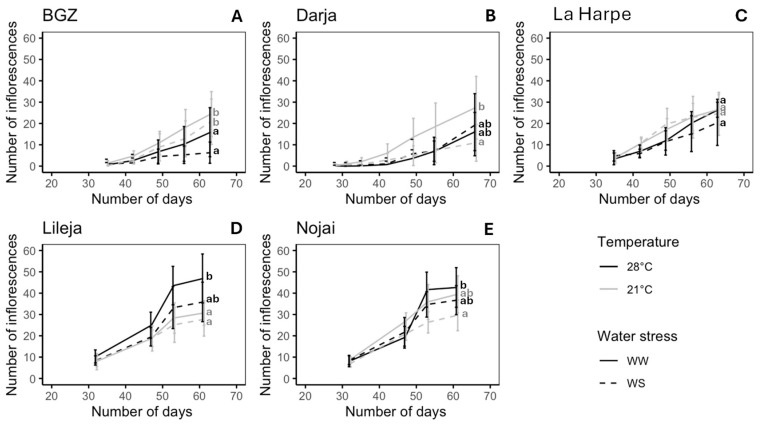
Inflorescence production of *F. esculentum* varieties subjected to two temperatures (21 °C vs. 28 °C) and water supply conditions (well-watered (WW) vs. water-stressed (WS)). Varieties: (**A**) Brabantse grijze zandboekweit (BGZ), (**B**) Darja, (**C**) La Harpe, (**D**) Lileja, and (**E**) Nojai. Data are means ± sd. Values followed by the same letter for the same variety were not statistically significant at the 5% level at the end of the experiment.

The number of inflorescences per plant ranged from 1 to 58, depending on the variety and the treatment (Figure 2). Inflorescence production was strongly affected by heat and slightly by drought in BGZ and Lileja, according to ANOVA2 (Table S2). It was only affected by drought in Nojai and by the interaction between both parameters in Darja (Table S2). La Harpe was the only variety to exhibit the same number of inflorescences, regardless of the treatment (Figure 2C). In BGZ (Figure 2A) and Darja (Figure 2B), the highest inflorescence production at the end of the experiment was observed at 21 °C WW, while the lowest was observed at 28 °C WS in BGZ (−74.1%) and at 21 °C WS in Darja (−60.2%). In Lileja (Figure 2D) and Nojai (Figure 2E), the highest inflorescence production at the end of the experiment was observed at 28 °C WW and the lowest at 21 °C WS (−25.8% in Lileja and −30.7% in Nojai).

#### 2.1.3. Floral Resources and Flower Fertility

**Figure 3:**
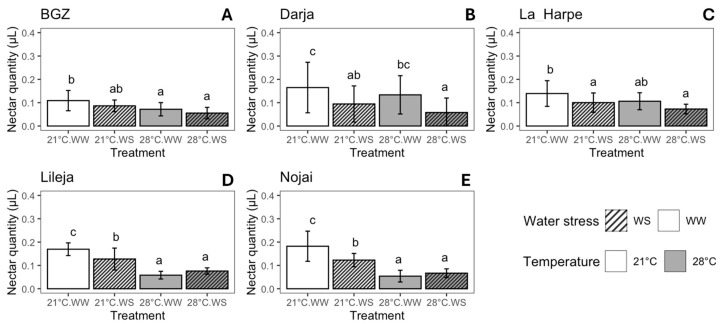
Nectar volume per flower (µL) for *F. esculentum* varieties subjected to two temperatures (21 °C vs. 28 °C) and water supply conditions (well-watered (WW) vs. water-stressed (WS)). Varieties: (**A**) Brabantse grijze zandboekweit (BGZ), (**B**) Darja, (**C**) La Harpe, (**D**) Lileja, and (**E**) Nojai. Data are means ± sd. Values followed by the same letter for the same variety were not statistically significant at the 5% level. Nectar was collected from a minimum of 5–10 flowers per morph for each variety and condition (details in Table S4).

**Figure 4:**
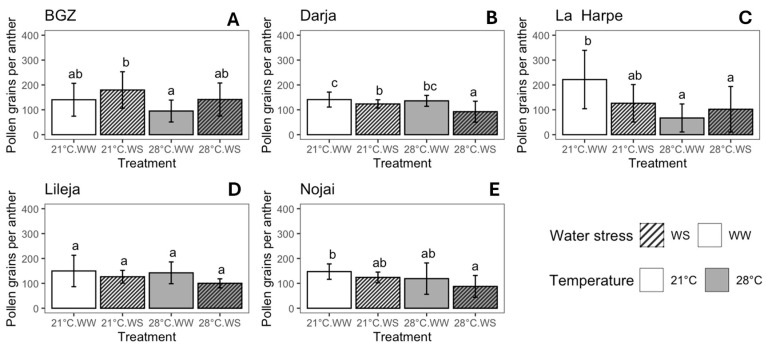
Number of pollen grains per anther for *F. esculentum* varieties subjected to two temperatures (21 °C vs. 28 °C) and water supply conditions (well-watered (WW) vs. water-stressed (WS)). Varieties: (**A**) Brabantse grijze zandboekweit (BGZ), (**B**) Darja, (**C**) La Harpe, (**D**) Lileja, and (**E**) Nojai. Data are means ± sd. Values followed by the same letter for the same variety were not statistically significant at the 5% level. Pollen was collected from a minimum of 5–10 flowers per morph for each variety and condition (details in Table S4).

Nectar and pollen production were evaluated, as they are key attractants for pollinators (Figure 3 and Figure 4, Table S3). Measurements of floral fertility, namely pollen viability and stigma receptivity, were also analyzed (Table 2 and Table S3). These parameters were evaluated separately for each flower morph (pin and thrum), and the details are presented in Table S4. The flower morph only affected the nectar volume per flower in Nojai and the pollen production in Darja (Tables S5 and S6); overall, both parameters were significantly higher in the pin morphs.

The mean nectar volume per flower ranged between 0.055 and 0.182 µL, and the highest volume was observed in plants grown at 21 °C WW, regardless of the variety (Figure 3). The nectar volume per flower decreased with high temperature and drought, although the effect was not similar in all varieties (Table S3). In BGZ flowers, nectar volume decreased regularly with both temperature increase and drought so that it was 45.4% lower in plants grown at 28 °C WS compared to those grown at 21 °C WW (Figure 3A, Table S3). In Darja, nectar volume decreased by 62.5% between 21 °C WW and 28 °C WS (Figure 3B, Table S3). In La Harpe, drought and high temperature both reduced the nectar volume (Table S3); the nectar volume per flower at 28 °C WS was 50% lower as compared with that at 21 °C WW (Figure 3C). The nectar volume of the Lileja and Nojai flowers was mainly affected by temperature, with a lower volume at 28 °C than at 21 °C, although it was also affected by drought at 21 °C (Figure 3D,E, Tables S3 and S5). For both varieties, the lowest nectar volume per flower was observed at 28 °C WW with a decrease of 65.7% and 70.4% compared to the results at 21 °C WW for Lileja and Nojai, respectively.

While the volume of nectar was affected by stress, its sugar concentration can also vary with the environment. The sugar concentration of nectar was only measured for Darja. It was 26.5% ± 4.5% at 21 °C WW, 15.6% ± 2.2% at 21 °C WS, 10.8% ± 6.7% at 28 °C WW, and 5.3 %± 2.3% at 28 °C WS, decreasing with both temperature increase and drought (Table S3). The sugar concentration in the nectar was also higher in the thrum than pin flowers, except at 21 °C WW (Tables S4 and S5).

The mean number of pollen grains per anther ranged from 67 to 222, depending on the variety and treatment (Figure 4). Here again, the effect of drought and high temperatures depended on the variety (Table S3). Pollen production increased with drought and slightly decreased with temperature in BGZ, while it decreased under both stresses and their combination in Darja (Figure 4A,B, Table S3). In La Harpe, pollen production decreased with temperature, and the effect of drought depended on the temperature (Figure 4C, Table S3). Pollen production was slightly affected by temperature in Nojai (Table S3), with a significant decrease between 21 °C WW and 28 °C WS (Figure 4E). Lileja was the variety whose pollen production was least affected by drought and heat (Figure 4D), although ANOVA2 revealed a low but significant decrease with drought (Table S3). As a result, the highest pollen production was observed at 21 °C WS in BGZ (Figure 4A) and at 21 °C WW for the other varieties (Figure 4B–E), and the lowest pollen production was observed at 28 °C WW for BGZ and La Harpe (Figure 4A,C) and at 28 °C WS for Darja (Figure 4B), Lileja (Figure 4D), and Nojai (Figure 4E). The decrease between the highest and lowest production ranged from 33.1% in Lileja to 69.6% in La Harpe.

Compared with pollen production, pollen viability was less affected by stress conditions (Table 2 and Table S3). It was only affected by high temperatures in Lileja and Nojai, with a decrease of 2–3% between 21 °C and 28 °C, but it was not affected by drought, regardless of the variety (Table 2).

Stigma receptivity was more sensitive to high temperatures and drought than was pollen viability. The highest stigma receptivity was observed at 21 °C WW for BGZ, Darja, and Lileja and at 21 °C WS for La Harpe and Nojai, with more than 75% of the analyzed flowers exhibiting receptive stigmata (Table 2). For most of the varieties, the lowest stigma receptivity was observed at 28 °C WS, with less than 55% of receptive stigmata, with the exception of Darja and Lileja, which showed the lowest stigma receptivity at 28 °C WW. Darja was the most affected variety, with only 8% of the flowers showing receptive stigmata at 28 °C WW, while La Harpe was the least affected variety, since more than 55% of the stigmata were receptive, regardless of the treatment (Table 2).

### 2.2. Impact of Drought and High Temperature on Buckwheat–Pollinator Interactions

For each variety, 3 to 5 plants per treatment at the flowering stage were transferred outside the greenhouses to observe the insect visiting behavior. Table 3 summarizes the number of inflorescences and open flowers per plant, as well as the total time of insect observation and the number of insects visiting the flowers. Statistical comparisons were not performed for these parameters due to the low number of plants. For BGZ, La Harpe, Lileja, and Nojai, three 10-min observation sessions were performed per treatment, yielding a total observation time of 120 min per variety. The different treatments of the same variety were observed at the same time under the same conditions to enable comparison. For Darja, all the plants from the different treatments were observed together during the different sessions, so the time given corresponds to the total observation time for all the treatments.

Except for Darja, the number of open flowers per plant was higher at 21 °C WW compared to that at the other conditions (Table 3). For Darja, plants grown at 28 °C WW displayed 3.9% more flowers in anthesis than those grown at 21 °C. For most varieties, the number of open flowers per plant decreased under stress conditions, and plants grown at 28 °C WS showed the lowest number of flowers at anthesis, regardless of the variety.

The number of insects visiting the flowers was affected by the plant-growing conditions (Table 3). For BGZ, more insects visited plants grown at 21 °C WW than at the other growing conditions. For Darja, the number of visiting insects was low and similar, regardless of the plant treatment. For La Harpe, the number of visiting insects was lower at 28 °C compared to that at 21 °C. For Lileja, the highest number of insects was observed on plants grown at 21 °C WS and for Nojai, at 28 °C WW. In general, the number of insects visiting the flowers of plants grown at 28 °C WS was lower than for the other treatments. Given the low number of insects visiting the flowers, the data from the different varieties will be compiled (see below) for further analysis of insect diversity and insect visiting behavior.

The flower visit rate provides information on the percentage of open flowers visited by insects per hour. It varied between 0 and 1.17, depending on the treatments and varieties. Nojai was the variety showing the highest flower visit rate at 21 °C WW. For BGZ and Lileja, the highest flower visit rate was for plants treated with only drought stress (21 °C WS), and for Darja and La Harpe, it was the highest for plants grown at 28 °C WS. However, the lowest flower visit rate was observed at 28 °C WS for BGZ, Lileja, and Nojai (Table 3).

#### 2.2.1. Insect Diversity

**Figure 5:**
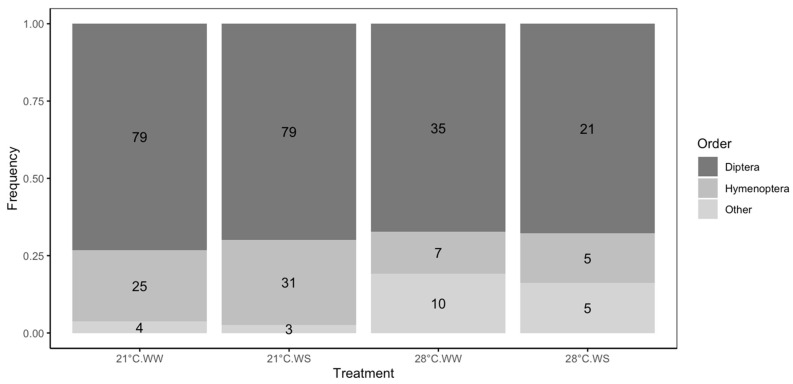
Diversity of pollinators visiting flowers of *F. esculentum* subjected to two temperatures (21 °C vs. 28 °C) and water supply conditions (well-watered (WW) vs. water-stressed (WS)). Flower visitors were categorized in three groups: Diptera (black), Hymenoptera (dark grey), and others (light grey). Numbers in the rectangles represent the number of insects observed for each order and treatment.

The main insects visiting *F. esculentum* flowers were Diptera (69.4%), followed by Hymenoptera (22.3%). Hoverflies accounted for about 69% of the observed Diptera, and *Apis mellifera*, solitary bees, and *Bombus* spp. accounted for, respectively, about 38%, 33%, and 29% of the observed Hymenoptera (Figure S2). In total, 108 insects were observed on flowers grown at 21 °C WW, 113 on flowers grown at 21 °C WS, 52 on flowers grown at 28 °C WW, and 31 on flowers grown at 28 °C WS (Figure 5). The number of insects visiting the flowers was affected by the plant-growing conditions and decreased with temperature (χ^2^ = 36.23, *p* < 0.0001.)

The proportions of the different insects also varied according to the treatment (χ^2^ = 22.79, *p* = 0.0008, Figure 5). The proportions of Diptera and mainly Hymenoptera were higher at 21 °C than at 28 °C, while the proportions of other insects were higher at 28 °C. Indeed, between 67.3% and 73.8% of visitors were Diptera at 21 °C compared with 67% at 28 °C, and between 22.4% and 27.4% were Hymenoptera at 21 °C compared with 13.1% to 16.1% at 28 °C. However, less than 3% of the visitors belonged to other orders at 21 °C compared with more than 16% at 28 °C.

#### 2.2.2. Insect Behavior

The visiting behavior of the insects was characterized based on the visiting time, the number of visited flowers, and the time spent on these flowers for the four plant treatments (Table 4). Flower visitors spent the same amount of time per visit to the plot, regardless of the taxonomic order or the treatment (Table 4 and Table S7). However, the time spent per flower was not the same for all insects and conditions, especially for Hymenoptera (Tables S7 and S8). Hymenoptera visited flowers of plants grown at 28 °C WS five times longer than the flowers grown under other conditions; such a difference was not observed for the other insects (Table 4). Consequently, the number of flowers visited per visit and per inflorescence differed between treatments for the Hymenoptera but not for the other insect groups (Tables S7 and S8). The number of flowers visited per insect was impacted by drought (F_1,63_= 4.63, *p* = 0.04), and the number of flowers visited per inflorescence was affected by the interaction between temperature and drought (F_1,63_ = 3.86, *p* = 0.05, Table S8) for Hymenoptera, but differences were not discriminated by post hoc tests due to the low *p*-values (Table 4).

### 2.3. Correlation Between Pollinator Behavior and Floral Resources

**Figure 6:**
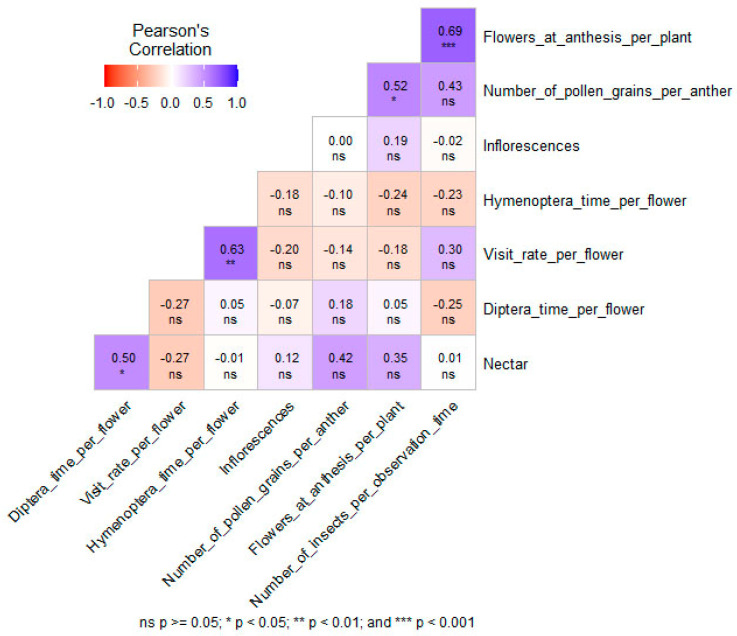
Pearson’s correlation matrix between pollinator visiting parameters and floral display and resource parameters in *F. esculentum* plants subjected to two temperatures (21 °C vs. 28 °C) and water supply conditions (well-watered (WW) vs. water-stressed). *p*-values are represented through four symbols: ns—*p* ≥ 0.05, * —*p* < 0.05, ** —*p* < 0.01, and *** —*p* < 0.001.

To determine whether the differences observed between treatments for the pollinator visiting behavior could be explained by differences in floral display and floral resources, a Pearson’s correlation matrix was elaborated (Figure 6). A total of 4 out of 28 correlations were significant and positive; no significant negative correlations were observed. The number of insects visiting the flowers per session was positively correlated with the number of open flowers per plant (R = 0.69, *p* < 0.001). The number of pollen grains per anther was also positively correlated with the number of flowers in anthesis per plant (R = 0.52, *p* < 0.05). Regarding the insect behavior, the time spent per flower by Diptera increased with the nectar volume per flower (R = 0.50, *p* < 0.05). The flower visit rate was also positively correlated with the time spent by the Hymenoptera on the flowers (R = 0.63, *p* < 0.01).

## 3. Discussion

### 3.1. The Impact of Drought and High Temperature on the Vegetative and Reproductive Growth Is Not Uniform Between the Different Buckwheat Varieties

Analyzing different varieties cultivated across various years makes the interpretations between the different stress conditions difficult due to the environmental variations. Therefore, it was not possible to make inter-varietal comparisons.

Leaf production did not follow the same pattern between the different stress conditions and the varieties (Figure 1). The number of leaves was stimulated by high temperature for two of the varieties (Figure 1C,D). An increase in leaf production with high temperature was also observed in *Arabidopsis thaliana*, *Solanum lycopersicum,* and *Borago officinalis* [60,61,62,63]. However, two other varieties produced the same number of leaves, regardless of the stress condition (Figure 1B,E), and one variety exhibited fewer leaves under drought conditions (Figure 1A). Previous results showed that heat tended to increase leaf production in common buckwheat, while drought had less of an impact [57,64,65]. Martínez-Goñi et al. also reported that common buckwheat was slightly affected by drought and developed drought avoidance mechanisms [66]. The differences observed between varieties suggest that the response to abiotic stress could be partly genotype-dependent in common buckwheat. Tryhub et al. showed that the ecological plasticity of buckwheat varieties could depend on the geographical origin, and that modern buckwheat varieties may display lower adaptability [66,67]. Buckwheat varieties that were domesticated under similar conditions indeed display similar morphological and physiological traits [66,67].

The impact of heat and drought on reproductive growth also varied among varieties. Although the flowering time was not strongly affected by the stress condition, the inflorescence production was either boosted by high temperatures or reduced by drought (Figure 2), even though the response could depend on the variety. These results are in accordance with the observations of Aubert et al. [57,64]. Aubert et al. also showed that heat and drought could have opposite effects on inflorescence production in Tartary buckwheat [68]. Chen et al. reported that maintaining flowering initiation during stress is a sign of stress tolerance and allows for the maintenance of reproduction [69]. It was proposed that, under stressful conditions, buckwheat produced more flowers to offset the lower number of flowers at anthesis [41,49,70]. We indeed observed a reduced number of open flowers with stress, mainly under combined heat and drought conditions.

From a global perspective, the impact of high temperature and/or drought on the growth of the different varieties was not uniform. Throughout all the experiments conducted over the years, the plants survived until the end of the experiment, showing an initial sign of tolerance to the different stresses. However, the resistance was correlated with the genotype of the plant. Varieties like Lileja increased their vegetative and reproductive growth under stress conditions, whereas other varieties like La Harpe tolerated the stress and did not modify their growth. Since the tolerance to drought and high temperature is variety-dependent, screening a large number of varieties under both stresses and their combination might be a good opportunity to determine tolerant and sensitive varieties and investigate their morphological and physiological differences in order to identify traits linked to stress tolerance in buckwheat.

### 3.2. Floral Resources and Female Fertility Are Negatively Affected by Drought and/or High Temperature

Floral resources were negatively affected by high temperatures and drought. Nectar volume was reduced by these stresses, regardless of the variety (Figure 3), as observed in other studies and species [52,71,72,73,74]. Indeed, these authors observed a decrease in nectar production under drought conditions, and that temperatures above 25 °C decrease nectar production in buckwheat [52,71,72]. Moreover, some authors reported that nectar production was genotype-dependent in buckwheat [51,75]. In our study, direct genotype comparisons were precluded, since the observations were performed over different years; however, the nectar volume per flower remained within the range of 0.1 to 0.2 µL under control conditions and exhibited a similar decreasing trend under stress conditions. The volume of nectar we observed per flower was in accordance with that observed in other studies [52,76]. We also measured the nectar sugar concentration in Darja and observed that it decreased with high temperatures and drought. In contrast to our results, Rering et al. did not observe a difference in total sugar concentration of the nectar in drought-stressed buckwheat plants, but they observed that drought decreased the proportion of sucrose in the nectar [52]. Heat and drought could have variable or no effects on nectar sugar concentrations, depending on the species and the study [52,73,77]. Moreover, our observations of nectar production may differ from those under natural conditions since they were performed under greenhouse conditions, and we cannot exclude possible bias, i.e., that nectar measurement can be influenced by the greenhouse humidity [77].

The number of pollen grains per anther is low in buckwheat compared to other species and was around 60–220 in our study, as previously observed [64]. We observed that stress affected pollen production, although the effect was variety-dependent (Figure 4). Aubert et al. observed that high temperatures increased pollen production, while drought reduced it [57,64]. Pollen production was affected by stress in other species such as *Collinsia heterophylla* [78].

Buckwheat is known to possess a high pollen viability and stress-sensitive female organs [53,79], while in most species, the male organs are more affected by stress than are the female organs [60,63,80,81,82,83]. We indeed observed that the pollen viability remained high, regardless of the variety (Table 2). However, stigmata receptivity decreased under stress conditions and more particularly, under combined heat and drought stress (Table 2).

### 3.3. Drought and High Temperature Affect Plant–Pollinator Interaction

Most of the flower visitors to buckwheat flowers in this study were Diptera (Figure 5), while Hymenoptera, such as *Apis mellifera* and *Apis cerana*, were also observed as the main pollinators in other studies [48,49]. Kim et al. also observed that buckwheat was mainly visited by Diptera [84]. Observations were performed on a limited number of plants in this study compared to the number present in a buckwheat field. It would thus be possible that Diptera were more attracted than Hymenoptera due to the limited number of plants. Additionally, it is not excluded that plants grown in a greenhouse exhibit some differences compared to field plants and thus, attract different pollinators.

Plants subjected to drought and/or high temperature were less visited by insects than were the control plants, and plants subjected to combined stress were the least visited (Table 3). Moreover, heat-stressed plants were less visited by Hymenoptera, while the proportion of Diptera visiting the flowers remained approximately constant, regardless of the stress condition (Figure 5). Drought, high temperature, and specifically, their combination, decreased the number of open flowers per plant, as well as the floral rewards, which could explain the lower attractivity for insects. Heat- and drought-stressed plants were also less visited by insects in *Borago* officinalis [60]. Floral display and floral rewards are indeed key elements affecting pollinator abundance and activity [85]. Nectar provides the main sugar source for insect pollinators, while pollen is the main source of polypeptides, amino acids, and phytosterols [4,86]. Therefore, modifications of floral traits and floral rewards might alter the attractiveness of flowers to pollinators [87,88]. Rering et al. also observed that drought stress affected floral traits and pollinator attraction in buckwheat, reducing plant reproductive success [52]. However, we observed that the flower visit rate was sometimes higher under stress conditions than under control conditions (Table 3), although this observation depended on the stress and the variety. Since the number of open flowers was lower in stressed plants, the chance of each individual flower to be visited was higher, suggesting that the reproductive success at the flower level could be maintained. The decrease in seed set at the plant level could thus be more explained by a reduced number of open flowers than by changes in flower visitation, as observed in *Sinapis arvensis* under drought stress [89].

Although the number of insects visiting stressed plants decreased, their visiting behavior was not affected, with the exception of Hymenoptera, which spent more time on the flowers of plants subjected to combined stress conditions (Table 4). Other studies reported that Hymenoptera spent more time on stressed than on non-stressed flowers [90], while others reported that the visitation time was higher on non-stressed than on stressed flowers [89], or that the time spent on individual flowers was not affected by stress [3]. Insect visiting behavior could be affected by several factors, explaining why the effect of stress on insect visitation rate and visitation behavior is non-linear and complex [3]. Moreover, the impact could depend on the type of insect, as it is known that foraging behavior may differ between Hymenoptera and Diptera and even between species [91,92]. In this study, a correlation was found between the volume of nectar and the time spent by Diptera per flower (Figure 6). These results could be linked to the tendency of Diptera to spend less time on stressed plants (Table 4), since they produced less nectar (Figure 3). However, this correlation must be interpreted with caution, because while the volume of nectar can affect visitor behavior, the flower’s sugar concentration and the total quantity of nectar sugar per flower are key factors in attracting visitors [89]. However, intrinsic pollinator behavior does not change with the plant’s stress conditions [89]. Nonetheless, pollinators will always choose non-stressed plants if given the choice between stressed or non-stressed types [3].

## 4. Conclusions

Overall, our results show that common buckwheat was affected by drought and high temperatures, and that the impact of these conditions depended on the variety of the plant. However, the stress combination had the strongest effect, regardless of the variety. Reproductive growth was also more affected than was vegetative growth. In particular, the number of open flowers and the floral rewards decreased with stress, negatively impacting the number of insects visiting the plants. Diptera were the most encountered flower visitors, and their frequency remained consistent across stress conditions. However, the frequency of Hymenoptera visiting flowers was lower for stressed than for non-stressed plants. The visitation behavior did not change between stressed and non-stressed flowers, except for the behavior of Hymenoptera, which spent more time on flowers subjected to combined stress. The manner in which these modifications in plant–pollinator interactions affect buckwheat reproductive success and seed set remain to be investigated.

## 5. Materials and Methods

### 5.1. Plant Material and Growth Conditions

Five commercial varieties of *Fagopyrum esculentum* were cultivated in this study, and their origin is described in Table S1. The plants were grown under greenhouse conditions at the SEFY platform (UCLouvain, Louvain-la-Neuve, Belgium) during independent experiments between 2018 and 2023. All varieties were not grown at the same time, explaining why the growth and reproductive parameters are presented independently for each variety.

The seeds were sown in peat compost (DCM, Amsterdam, The Netherlands) and about 10 days later, the seedlings were transplanted individually into 2 L pots containing the same peat compost. The plants were grown under greenhouse conditions (relative humidity of 65% ± 7%, 21 °C/19 °C day/night temperature, and a 14 or 16 h photoperiod, depending on the experiment). LED LumiGrow lights (650 W, red-blue) to obtain a minimum mean light intensity of 150 µmol m^−2^·s^−1^ provided additional lighting, when necessary. Two weeks after sowing, half of the plants were moved to another greenhouse compartment under the same conditions but with a 28 °C/26 °C day/night temperature, and four treatments were applied: 21 °C/19 °C well-watered (40–50% soil humidity; 21 WW), 21 °C/19 °C water-stressed (20–25% soil humidity; 21 WS), 28 °C/26 °C well-watered (40–50% soil humidity; 28 WW), and 28 °C/26 °C water-stressed (20–25% soil humidity; 28 WS). Watering was performed on Monday, Wednesday, and Friday for the WW plants and on Monday and Friday for the WS plants. Soil humidity was monitored three times a week using a ProCheck sensor handheld reader (Decagon Devices, Pullman, WA, USA). The temperature and relative humidity in the greenhouse compartments were monitored every ten minutes. The experiments lasted a maximum of 63 days, with a minimum of 10 plants per condition and variety. The temperature and air and soil relative humidity of the different experiments are shown in the Supplemental Materials (Figure S1).

### 5.2. Vegetative and Reproductive Growth Parameters Assessment

The total number of leaves and inflorescences per plant were monitored on a weekly basis for 10 plants per condition and variety. A leaf was counted once it was fully unfolded, whereas an inflorescence was counted when all the flower buds turned white. The node of the first inflorescence was also noted to estimate the flowering time (nodes were counted acropetally, and the cotyledonary node was disregarded).

### 5.3. Flower Fertility and Floral Resources Assessment

The number of pollen grains per anther, the pollen viability, the stigma receptivity, and the nectar volume were evaluated on 10 flowers per condition and morph for Darja, Lileja, and Nojai and on 5 flowers per condition and morph for BGZ and La Harpe.

For the quantification of pollen grains per anther, the flowers were harvested at the flower bud stage and were stored in FAA (5 mL formaldehyde 35%, 5 mL glacial acetic acid, 90 mL ethanol 70% *v*/*v*). For each flower, two anthers were sampled; each was crushed on a microscope slide to extract the pollen grains, and 10 μL of Alexander dye were added to stain the pollen grains (Alexander., 1969). The number of pollen grains per slide was counted under a light microscope (Polyvar, Reichert-Jung, Depew, NY, USA), according to the methods of Ref. [93].

For pollen viability, fresh pollen was collected in a small Petri dish by shaking the inflorescences; the pollen was then transferred to a microscope slide, 10 μL of Alexander dye was added, and the percentage of pollen viability was assessed under a light microscope (Polyvar, Reichert-Jung, Depew, NY, USA) by analyzing a minimum of 200 pollen grains per slide. The pollen was considered viable (presence of cytoplasm) when a red coloration appeared, while it was considered non-viable (absence of cytoplasm) when the coloration turned blue. Ten microscope slides were analyzed per condition and variety.

Stigma receptivity was assessed on fully opened flowers by detecting the peroxidase activity of the stigma, as described by Dafni and Maués (1998): reddish-brown color on the surface received a score of 0 (no color = no receptive stigma) or 1 (reddish-brown color = receptive stigma) [94]. Flower anthesis lasts for one day in buckwheat [40], and stigma receptivity was assessed at the beginning of the afternoon.

Nectar was collected on a minimum of 10 flowers per condition and variety (same number of pin and thrum flowers) on the fifth or sixth inflorescences and on a minimum of five different plants per condition, morph, and variety. Using a 0.5 μL micro-capillary, the nectar was collected inside the corolla at the nectary level. The nectar volume per flower was calculated by dividing the amount of the nectar in the micro-capillary by the total length of the micro-capillary, according to the methods of [95]. All measurements were performed between 3 p.m. and 4 p.m. The sugar concentration in the nectar was also measured in var. Darja using a low-volume hand refractometer (Eclipse Handheld Refractometer; Bellingham & Stanley Ltd., Tunbridge Wells, UK) and is expressed as percentages of sucrose per nectar mass (*w*/*w*). Due to the low volume of nectar per flower, 1 µL or 5 µL micro-capillaries were filled with the nectar of several flowers, and one drop was placed on the refractometer to determine the average sugar concentration. Six readings were obtained per variety, morph, and condition.

### 5.4. Insect Visitation and Behavior Assessment

To evaluate the impact of the growing conditions on the plant attractivity to visiting insects, three to five plants per condition and variety (after at least 50 days of treatment) were placed in the garden just outside the greenhouse (50°39′55.3″ N, 4°37′09.9″ E) to observe insect visitation and behavior. The plants were taken back in the greenhouse at the end of each day after the observation sessions. Each observation session lasted 10 min and covered a “plot” of three plants, and at least three observation sessions were performed for each condition and variety. The number of plants per “plot” was chosen so that all the plants could be tracked in the same field of view, and so that all the insects visiting the flowers in the “plot” could be monitored at the same time. The same proportion of pin and thrum plants was used for the different conditions of the same variety, so that the flower morph did not affect the insect visit results. Variety Darja, however, was observed for a longer duration because all the plants from the different conditions were put outside at the same time. For each variety, the observations were performed for the different conditions on the same days under the same weather conditions. Flower visitations were recorded on sunny days between 10:00 h and 16:00 h. At the beginning of each observation session, meteorological parameters (nebulosity, wind force, temperature) and plant parameters of the “plot” (number of plants, number of inflorescences per plant, number of flowers in anthesis per plant) were recorded. Visitors were classified as honeybees (*Apis mellifera*), bumblebees, solitary bees, hoverflies, other Diptera (Diptera excluding Syrphidae), and other minor visitors. Visiting insects were tracked from the moment they visited a flower in the “plot” until they left the “plot”, and during the visit of an insect within the “plot”, the type of insect; the number of flowers, inflorescences, and plants visited; as well as the visit time per flower, and the total visit time within the “plot”, were noted. The flower visitation rates were calculated as the ratios between the number of flowers visited by insects in a plot per hour and the total number of open flowers in the plot.

### 5.5. Statistical Analyses

Statistical analyses were conducted in R studio (Version 4.3.3). The homoscedasticity and normality of the data were assessed using the Levene, Bartlett, and Shapiro–Wilk tests. Data were transformed (square root or logarithm) when required to satisfy the normality of the residuals. Each variety was analyzed individually, since the year of culture and the environmental conditions fluctuated throughout the different experiments. ANOVA2 models were defined to evaluate the effects of the temperature, the water stress, and their interactions on the characteristics of the plants and the floral resources for each variety. For floral resources, ANOVA3 models were used to evaluate the effects of the morph, the temperature, the water stress, and their interactions for each variety. ANOVA3 models were also used to evaluate the impact of temperature, water stress, insect order, and their interactions on the insect visiting behavior. Multiple comparisons tests using the Tukey (for balanced data) and Šídák (for unbalanced data) methods were performed for ANOVA analysis, with significant values. Chi-square tests were performed to compare the number of insects according to the treatment and the frequency of Diptera, Hymenoptera, and other insects, according to the treatment. A Pearson’s correlation matrix (with Bonferroni-corrected *p* values) was performed between insect visiting parameters, floral display, and floral resources. If not indicated otherwise, data were presented as means ± sd. The statistical data are presented in Tables S2, S3, and S5–S8 (Supplementary Materials).

## Figures and Tables

**Table 1:** Node of the first inflorescences for *F. esculentum* varieties subjected to two temperatures (21 °C vs. 28 °C) and water supply conditions (well-watered (WW) vs. water-stressed (WS)). Data are means ± sd. Values followed by the same letter for the same variety were not statistically significant at the 5% level.

	21 °C WW	21 °C WS	28 °C WW	28 °C WS
BGZ	6.60 ± 1.07 a	7.20 ± 2.04 a	7.70 ± 1.06 a	7.30 ± 2.98 a
Darja	5.50 ± 0.97 a	5.78 ± 0.97 a	5.10 ± 0.99 a	6.00 ± 0.94 a
La Harpe	4.20 ± 0.42 b	4.70 ± 0.67 ab	4.60 ± 0.52 ab	5.20 ± 1.03 a
Lileja	5.11 ± 0.33 a	4.89 ± 0.33 a	5.33 ± 0.71 a	5.00 ± 0.00 a
Nojai	5.00 ± 0.58 a	5.00 ± 0.58 a	5.00 ± 0.89 a	4.71 ± 0.76 a

**Table 2:** Pollen viability and stigmata receptivity for *F. esculentum* varieties subjected to two temperatures (21 °C vs. 28 °C) and water supply conditions (well-watered (WW) vs. water-stressed (WS)).

Variety *	21 °C WW	21 °C WS	28 °C WW	28 °C WS
Pollen viability (%)				
BGZ	77.72 ± 21.64 a	86.15 ± 11.42 a	78.85 ± 19.22 a	87.45 ± 7.18 a
Darja	95.59 ± 6.07 a	94.72 ± 3.76 a	95.77 ± 4.13 a	97.63 ± 2.45 a
La Harpe	91.50 ± 5.47 a	90.97 ± 7.19 a	86.77 ± 8.36 a	90.68 ± 5.31 a
Lileja	98.20 ± 0.98 b	97.68 ± 1.55 b	94.85 ± 2.49 a	95.90 ± 1.73 a
Nojai	98.68 ± 0.94 b	98.50 ± 0.93 b	96.28 ± 3.05 a	96.33 ± 2.63 a
Stigmata receptivity (%)				
BGZ	85	55	60	40
Darja	90	80	8	33
La Harpe	60	75	65	55
Lileja	90	60	35	45
Nojai	65	90	75	25

* Mean values and their standard deviation which are followed by the same letter for the same variety were not statistically significant at the 5% level. Pollen viability and stigma receptivity were analyzed using a minimum of 5–10 flowers per morph for each variety and condition (details in Table S4).

**Table 3:** Insect visitation parameters for *F. esculentum* varieties subjected to two temperatures (21 °C vs. 28 °C) and water supply conditions (well-watered (WW) vs. water-stressed (WS)). For open flowers and inflorescences per plant, data are means ± sd.

Treatment	Observed Plants (n)	Observation Time (min)	Open Flowers per Plant	Inflorescences per Plant	Number of Insects	Flower Visit Rate/h
BGZ						
21 °C WW	5	30	268.93 ± 23.89	26.89 ± 2.39	47	0.29
21 °C WS	5	30	102.98 ± 32.85	20.60 ± 6.57	42	0.62
28 °C WW	5	30	135.46 ± 36.71	16.93 ± 4.59	15	0.10
28 °C WS	5	30	64.00 ± 00.00	8.00 ± 0.00	2	0.04
Darja						
21 °C WW	3	261 *	34.33 ± 7.09	16.67 ± 5.77	5	0.09
21 °C WS	3	261 *	27.33 ± 6.66	17.67 ± 3.79	7	0.23
28 °C WW	3	261 *	35.67 ± 24.50	27.00 ± 4.58	6	0.05
28 °C WS	3	261 *	6.33 ± 4.04	7.67 ± 2.31	6	1.17
La Harpe						
21 °C WW	5	30	217.00 ± 23.50	31.00 ± 3.36	42	0.30
21 °C WS	5	30	128.11 ± 24.93	25.79 ± 4.89	48	0.50
28 °C WW	5	30	87.60 ± 9.61	21.9 ± 2.40	20	0.38
28 °C WS	5	30	27.55 ± 9.50	13.77 ± 4.75	22	0.98
Lileja						
21 °C WW	3	30	146.01 ± 5.91	48.67 ± 1.97	7	0.05
21 °C WS	3	30	141.52 ± 7.56	35.38 ± 1.89	11	0.25
28 °C WW	3	30	128.58 ± 9.06	42.86 ± 3.02	3	0.19
28 °C WS	3	30	96.51 ± 37.98	32.17 ± 12.66	0	0.00
Nojai						
21 °C WW	3	30	148.68 ± 8.28	35.40 ± 1.97	7	0.28
21 °C WS	3	30	78.08 ± 4.03	24.40 ± 1.26	5	0.13
28 °C WW	3	30	83.50 ± 11.28	38.50 ± 5.20	8	0.23
28 °C WS	3	30	50.88 ± 00.49	39.14 ± 0.38	1	0.10

* All treatments were observed concurrently; therefore, it was not possible to evaluate the time interval per treatment.

**Table 4:** Visiting behavior of pollinators observed on flowers of *F. esculentum* plants subjected to two temperatures (21 °C vs. 28 °C) and water supply conditions (well-watered (WW) vs. water-stressed (WS)).

Conditions	Number of Insects	Time per Visit to the Plot (s) *	Time per Flower Visit (s) *	Flowers Visited per Insect *	Visited Flowers per Inflorescence *
Diptera					
21 °C WW	79	96.61 ± 106.36 a	15.36 ± 31.56 a	9.67 ± 10.34 a	2.59 ± 1.39 a
21 °C WS	35	65.80 ± 100.62 a	11.46 ± 12.63 a	6.83 ± 8.39 a	2.14 ± 0.89 a
28 °C WW	79	77.75 ± 68.26 a	9.56 ± 8.12 a	9.57 ± 8.84 a	2.39 ± 1.08 a
28 °C WS	21	60.9 ± 91.47 a	7.88 ± 5.64 a	8.19 ± 12.34 a	2.17 ± 0.79 a
Hymenoptera					
21 °C WW	24	97.29 ± 113.15 a	7.18 ± 5.62 a	16.84 ± 22.38 a	3.09 ± 3.66 a
21 °C WS	7	36.29 ± 38.75 a	5.87 ± 4.39 a	5.86 ± 2.67 a	1.85 ± 0.42 a
28 °C WW	31	56.16 ± 58.35 a	8.04 ± 8.32 a	7.77 ± 7.31 a	2.00 ± 1.04 a
28 °C WS	5	128.60 ± 121.22 a	40.07 ± 32.76 b	3.20 ± 1.30 a	2.70 ± 0.84 a
Others					
21 °C WW	4	109.50 ± 138.19 a	7.19 ± 5.15 a	10.50 ± 9.61 a	7.81 ± 10.2 a
21 °C WS	10	109.60 ± 100.18 a	9.05 ± 6.18 a	16.50 ± 17.28 a	5.05 ± 5.68 a
28 °C WW	3	127.33 ± 112.38 a	6.90 ± 5.47 a	16.67 ± 16.50 a	3.30 ± 0.51 a
28 °C WS	5	33.60 ± 30.47 a	5.96 ± 3.87 a	5.60 ± 4.98 a	2.06 ± 0.77 a

* Data are means ± sd. Values followed by the same letter for the same order were not statistically significant at the 5% level.

## Data Availability

The data presented in this study are available in the text and Supplementary Materials. The data presented in this study are available upon request from the corresponding author.

## Supplementary Materials

The following supporting information can be downloaded at: https://www.mdpi.com/article/10.3390/plants14010131/s1, Figure S1: Environmental conditions during the experiment for each variety; Figure S2: Some insects visiting *Fagopyrum esculentum* flowers during the experiment. (A–C) Syrphidae (Diptera), (D–F) Hymenoptera; Table S1: Origin country and institution for each *F. esculentum* variety; Table S2: Results of statistical analyses (ANOVA2) for plant growth parameters for *F. esculentum*; Table S3: Results of statistical analyses (ANOVA2) for floral fertility and resources for *F. esculentum*; Table S4: floral resources and floral fertility parameters of *F. esculentum* plants grown under two temperatures (21 °C vs. 28 °C) and water supply conditions (well-watered (WW) vs. water-stressed (WS)), according to the variety and flower morph; Table S5: Results of statistical analyses (ANOVA2) for nectar parameters (nectar volume per flower and sugar concentration) for the different flower morphs of *F. esculentum*; Table S6: Results of statistical analyses (ANOVA3) for number of pollen grains per anther for the different flower morphs of *F. esculentum*; Table S7: Results of statistical analyses (ANOVA3) for the behavior of insects pollinating plants subject at two temperatures (21 °C vs. 28 °C) and water supply conditions (well-watered (WW) vs. water-stressed (WS)); Table S8: Results of statistical analyses (ANOVA2) for the behavior of Diptera, Hymenoptera, and other insects pollinating plants subjected to two temperatures (21 °C vs. 28 °C) and water supply conditions (well-watered (WW) vs. water-stressed (WS)).
