# Supplementary material for: Drought and High Temperatures Impact the Plant–Pollinator Interactions in Fagopyrum esculentum
Source: Plants (Basel). 2025 Jan 4;14(1):131. doi: 10.3390/plants14010131 (PMC11722719; doi:10.3390/plants14010131)
Supplement: Supplementary file 1 [file plants-14-00131-s001.zip › plants-3333145-supplementary.pdf]

Supplemental data

Table S1: Origin country and institution for each *F. esculentum* variety.

Variety	Origin country	Received by
Darja	Slovenia	I.Kreft / University of Ljubljana
La Harpe	France	Gired
Lileja	ND*	Cepicop
Nojai	ND*	Cepicop
Brabantse grijze zandboekweit (BGZ)	The Netherlands**	C. Zewen / Luxembourg

* ND: no information about the origin country
** old cultivated variety

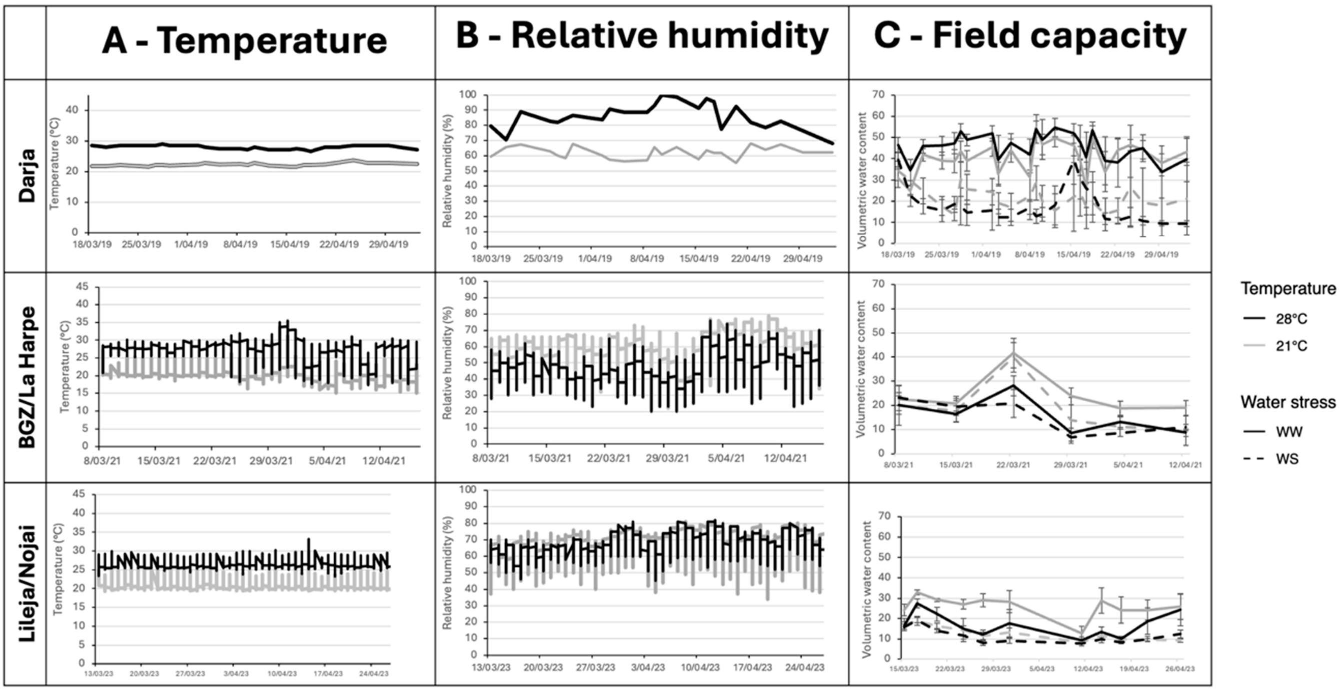

Figure S1: Environmental conditions during the experiment for each *F. esculentum* variety. (A) Temperature, (B) Relative humidity in the greenhouse. Grey: greenhouse at 21°C, Black: greenhouse at 28°C. (C) Soil water content in pots before watering subjected to 21 °C (grey lines) vs 28 °C (black lines) and well-watered (complete line) vs. water-stressed (dashed lines). Watering was performed Monday, Wednesday, and Friday for WW plants and Monday and Friday for WS plants.

Table S2: Results of statistical analyses (ANOVA2) of plant growth parameters for *F. esculentum*. Each variety was subjected to two temperatures (21 °C vs. 28 °C) and water supply conditions (well-watered (WW) vs. water stressed (WS)). * = P <0.05, **= P < 0.01 and *** = P < 0.001. Grey boxes represent significant P-values.

		**BGZ**		**Darja**		**La Harpe**		**Lileja**		**Nojai**	
**Number of Leaves**	Temperature	$F_{1,36}$= 6.47	**P = 0.01 ***	$F_{1,34}$ = 0.002	P = 0.96	$F_{1,36}$ = 2.71	P = 0.10	$F_{1,32}$ = 14.37	**P = 6.28*10^{-4} *****	$F_{1,23}$ = 1.09	P = 0.3
	Hydric stress	$F_{1,36}$ = 15.6	**P = 3.14*10^{-4} *****	$F_{1,34}$ 0.03	P = 0.87	$F_{1,36}$ = 27.5	**P = 6.97*10^{-6} *****	$F_{1,32}$ = 2.17	P = 0.15	$F_{1,23}$ = 3.50	P = 0.07 .
	Temperature : Hydric stress	$F_{1,36}$ = 0.88	P = 0.35	$F_{1,34}$= 8.18	**P = 7.19*10^{-3} ****	$F_{1,36}$ = 7.07	**P = 0.01 ***	$F_{1,32}$ = 0.631	P = 0.43	$F_{1,23}$ = 2.30	P = 0.14
**Number of inflorescences**	Temperature	$F_{1,36}$ = 13.86	**P = 6.71*10^{-4} *****	$F_{1,34}$ = 0.13	P = 0.73	$F_{1,36}$ = 0.60	P = 0.45	$F_{1,32}$ =17.29	**P = 2.25*10^{-4} *****	$F_{1,23}$ = 2.60	P = 0.12
	Hydric stress	$F_{1,36}$ = 4.57	**P = 0.04***	$F_{1,34}$ = 3.04	P = 0.09 .	$F_{1,36}$ =2.21	P = 0.14	$F_{1,32}$ = 5.67	**P = 0.02 ***	$F_{1,23}$ =6.71	**P = 0.02 ***
	Temperature : Hydric stress	$F_{1,36}$ = 0.94	P = 0.34	$F_{1,34}$ = 6.67	**P = 0.01 ***	$F_{1,36}$ = 0.54	P = 0.47	$F_{1,32}$ =1.85	P = 0.18	$F_{1,23}$ =0.40	P = 0.53
**Node of the first inflorescence**	Temperature	$F_{1,36}$ = 0.94	P = 0.34	$F_{1,34}$ = 0.07	P = 0.80	$F_{1,36}$ = 4.12	**P = 0.04 ***	$F_{1,32}$ = 1.39	P = 0.25	$F_{1,23}$ = 0.32	P = 0.58
	Hydric stress	$F_{1,36}$ = 0.03	P = 0.88	$F_{1,34}$ = 3.69	P = 0.06 .	$F_{1,36}$ = 6.15	**P = 0.02 ***	$F_{1,32}$ = 3.84	P = 0.06 .	$F_{1,23}$ = 0.26	P = 0.62
	Temperature : Hydric stress	$F_{1,36}$ = 0.65	P = 0.43	$F_{1,34}$ = 1.00	P = 0.32	$F_{1,36}$ = 0.05	P = 0.82	$F_{1,32}$ = 0.15	P = 0.70	$F_{1,23}$ = 0.28	P = 0.60

Table S3: Results of statistical analyses (ANOVA2) for floral fertility and floral resources for *F. esculentum*. Each variety was subjected to two temperatures (21 °C vs. 28 °C) and water supply conditions (well-watered (WW) vs. water stressed (WS)). * = P <0.05, **= P < 0.01 and *** = P < 0.001. Grey boxes represent significant P-values. ND = not determined.

		**BGZ**		**Darja**		**La Harpe**		**Lileja**		**Nojai**	
**Nectar quantity**	Temperature	$F_{1,76}$ = 18.72	**P = 4.54*10^{-5} *****	$F_{1,80}$ = 2.81	P = 0.09 .	$F_{1,76}$ = 13.20	**P = 5.06*10^{-4} *****	$F_{1,76}$ = 155.65	**P = <2.00*10^{-16} *****	$F_{1,76}$ = 114.04	**P = <2.00*10^{-16} *****
	Hydric stress	$F_{1,76}$ = 6.80	**P = 0.01 ***	$F_{1,80}$ = 15.85	**P = 1.50*10^{-4} *****	$F_{1,76}$ = 13.05	**P = 5.41*10^{-4} *****	$F_{1,76}$ = 3.50	P = 0.07 .	$F_{1,76}$ = 7.36	**P = 8.26 *10^{-3} ****
	Temperature : Hydric stress	$F_{1,76}$ = 0.00	P = 0.98	$F_{1,80}$ = 0.02	P = 0.89	$F_{1,76}$ = 1.50	P = 0.22	$F_{1,76}$ = 20.94	P = 7.94*10^{-5}	$F_{1,76}$ = 17.41	**P = 7.94*10^{-5} *****
**Sugar (%) in nectar**	Temperature	ND	ND	$F_{1,40}$ = 160.8	**P <0.0001 ***.**	ND	ND	ND	ND	ND	ND
	Hydric stress	ND	ND	$F_{1,40}$ = 64.1	**P <0.0001**	ND	ND	ND	ND	ND	ND
	Temperature : Hydric stress	ND	ND	$F_{1,40}$ = 6.70	**P = 0.0134 ***	ND	ND	ND	ND	ND	ND
**Number of pollen grains**	Temperature	$F_{1,36}$ = 4.35	**P = 0.04 ***	$F_{1,36}$ = 15.76	**P = 1.09*10^{-4} *****	$F_{1,36}$ = 10.30	**P = 2.79*10^{-3} ****	$F_{1,36}$ = 1.67	P = 0.21	$F_{1,36}$ = 5.63	**P = 0.02 ***
	Hydric stress	$F_{1,36}$ = 4.54	**P = 0.04 ***	$F_{1,36}$ = 43.39	**P = 6.32*10^{-10} *****	$F_{1,36}$ = 1.18	P = 0.28	$F_{1,36}$ = 6.22	**P = 0.017 ***	$F_{1,36}$ = 4.10	P = 0.05 .
	Temperature : Hydric stress	$F_{1,36}$ = 0.03	P = 0.86	$F_{1,36}$ = 7.94	**P = 5.45*10^{-3} ****	$F_{1,36}$ = 5.49	**P = 0.02 ***	$F_{1,36}$ = 0.53	P = 0.47	$F_{1,36}$ = 0.09	P = 0.77
**Pollen viability**	Temperature	$F_{1,20}$ = 0.04	P = 0.85	$F_{1,53}$ = 2.17	P = 0.15	$F_{1,20}$ = 0.84	P = 0.37	$F_{1,76}$ = 41.82	**P = 8.72*10^{-9} *****	$F_{1,76}$ = 23.36	**P = 6.84*10^{-6} *****
	Hydric stress	$F_{1,20}$ = 1.7	P = 0.20	$F_{1,53}$ = 0.90	P = 0.35	$F_{1,20}$ = 0.38	P = 0.54	$F_{1,76}$ = 0.44	P = 0.51	$F_{1,76}$ = 0.02	P = 0.90
	Temperature : Hydric stress	$F_{1,20}$ = 0.00	P = 0.99	$F_{1,53}$ = 1.48	P = 0.23	$F_{1,20}$ = 0.66	P = 0.43	$F_{1,76}$ = 3.95	P = 0.05 .	$F_{1,76}$ = 0.06	P = 0.81

Table S4: floral resources and floral fertility parameters of *F. esculentum* plants grown under two temperatures (21 °C vs. 28 °C) and water supply conditions (well-watered (WW) vs. water stressed (WS)) according to the variety and flower morph. ND = not determined.

Variety	Temperature	Stress	Flower morph	Nectar volume per flower (µl)	Sugar in nectar (%)	Number of pollen grains per anther	Stigma receptivity (%)	Pollen viability (%)
BGZ	21°C	WS	thrum	0,089 ± 0,022	ND	170,2 ± 88,28	50,00	80,75 ± 15,1
			pin	0,084 ± 0,03	ND	189,2 ± 64,14	100,00	91,56 ± 3,24
		WW	thrum	0,101 ± 0,045	ND	159,6 ± 85,65	70,00	62,1 ± 20,75
			pin	0,117 ± 0,043	ND	121,4 ± 39,74	50,00	93,34 ± 2,9
	28°C	WS	thrum	0,039 ± 0,023	ND	96,6 ± 45,29	30,00	85,5 ± 10,33
			pin	0,071 ± 0,014	ND	186,2 ± 53,44	70,00	89,4 ± 3,27
		WW	thrum	0,074 ± 0,022	ND	80,4 ± 29,81	50,00	70,7 ± 26,5
			pin	0,069 ± 0,037	ND	109,8 ± 54,17	73,30	86,99 ± 4,79
Darja	21°C	WS	thrum	0,091 ± 0,061	14.4 ± 2.6	112,4 ± 9,13	100,00	93,5 ± 3,98
			pin	0,098 ± 0,096	16.8 ± 0.9	134,7 ± 15,31	87,00	95,94 ± 3,41
		WW	thrum	0,161 ± 0,130	29.2 ± 3.0	119,2 ± 20,68	100,00	94,37 ± 7,32
			pin	0,168 ± 0,087	23.8 ± 4.1	161 ± 22,57	0,00	97,22 ± 4,81
	28°C	WS	thrum	0,064 ± 0,066	6.1 ± 1.0	77,6 ± 34,65	67,00	97,45 ± 2,52
			pin	0,073 ± 0,047	4.5 ± 3.0	107,3 ± 44,51	6,00	97,85 ± 2,52
		WW	thrum	0,174 ± 0,060	15.1 ± 7.0	126,4 ± 14,84	11,00	96,57 ± 4,39
			pin	0,093 ± 0,098	6.6 ± 2.8	145,6 ± 23,94	80,00	95,12 ± 3,99
La_Harpe	21°C	WS	thrum	0,106 ± 0,051	ND	87,8 ± 34,68	70,00	90,12 ± 10,71
			pin	0,095 ± 0,033	ND	164,8 ± 88,36	20,00	91,82 ± 3,5
		WW	thrum	0,139 ± 0,068	ND	238,8 ± 109,9	100,00	90,53 ± 7,56
			pin	0,14 ± 0,042	ND	204,6 ± 134,67	70,00	92,46 ± 3,85
	28°C	WS	thrum	0,072 ± 0,013	ND	83 ± 52,23	40,00	88,18 ± 4,46
			pin	0,073 ± 0,026	ND	121,4 ± 123,27	60,00	93,19 ± 5,63
		WW	thrum	0,096 ± 0,044	ND	57,8 ± 17,94	70,00	82,49 ± 9,65
			pin	0,120 ± 0,019	ND	76,6 ± 81,25	50,00	91,06 ± 5,14
Lileja	21°C	WS	thrum	0,150 ± 0,055	ND	126,6 ± 25,69	70,00	98,75 ± 0,68
			pin	0,105 ± 0,022	ND		100,00	96,6 ± 1,43
		WW	thrum	0,160 ± 0,027	ND	149,8 ± 63,18	80,00	97,6 ± 0,77
			pin	0,179 ± 0,025	ND		70,00	98,8 ± 0,79
	28°C	WS	thrum	0,073 ± 0,014	ND	100,1 ± 18,06	20,00	97,3 ± 1,09
			pin	0,079 ± 0,013	ND		30,00	94,5 ± 0,88
		WW	thrum	0,059 ± 0,015	ND	142,4 ± 43,81	40,00	96,25 ± 2,53
			pin	0,058 ± 0,019	ND		90,00	93,45 ± 1,54
Nojai	21°C	WS	thrum	0,131 ± 0,016	ND	123,8 ± 21,5	90,00	98,7 ± 0,63
			pin	0,115 ± 0,036	ND		90,00	98,3 ± 1,16
		WW	thrum	0,139 ± 0,036	ND	147,2 ± 30,99	80,00	98,25 ± 1,06
			pin	0,225 ± 0,059	ND		30,00	99,1 ± 0,57
	28°C	WS	thrum	0,075 ± 0,02	ND	87,7 ± 43,9	20,00	98,45 ± 1,14
			pin	0,058 ± 0,013	ND		60,00	94,2 ± 1,8
		WW	thrum	0,042 ± 0,031	ND	119,1 ± 63,03	90,00	98,2 ± 1,3
			pin	0,066 ± 0,007	ND		90,00	94,35 ± 3,11

Table S5: Results of statistical analyses (ANOVA2) for nectar parameters (nectar volume per flower and sugar concentration) for the different flower morphs of *F. esculentum*. Each variety was subjected to two temperatures (21 °C vs. 28 °C) and water supply conditions (well-watered (WW) vs. water stressed (WS)). * = P <0.05, **= P < 0.01 and *** = P < 0.001. Grey boxes represent significant P-values.

	**Nectar quantity (µl)**										**Sugar in nectar (%)**	
	**BGZ**		**Darja**		**La Harpe**		**Lileja**		**Nojai**		**Darja**	
**Temperature**	$F_{1,55}$ = 17.40	**P <0.0001 *** **	$F_{1,134}$ = 2.54	P = 0.1135	$F_{1,61}$ = 8.39	**P = 0.0052 ** **	**$F_{1,72}$ = 181.57**	**P <0.0001 *** **	$F_{1,72}$ = 172.81	**P <0.0001 *** **	$F_{1,40}$ = 160.86	**P <0.0001 *** **
**Hydric stress**	$F_{1,55}$ = 5.56	**P = 0.0219 * **	$F_{1,134}$ = 14.57	**P = 0.0002 *** **	$F_{1,61}$ = 12.90	**P = 0.0007 *** **	**$F_{1,72}$ = 1.07**	**P = 0.0473 * **	$F_{1,72}$ = 11.15	**P = 0.0013 ** **	$F_{1,40}$ = 64.12	**P <0.0001 *** **
**Flower morph**	$F_{1,55}$ = 1.34	P = 0.2516	$F_{1,134}$ = 1.73	P = 0.1903*	$F_{1,61}$ = 0.11	P = 0.7445	$F_{1,72}$ = 0.68	P = 0.4138	$F_{1,72}$ = 7.29	**P = 0.0086 ** **	$F_{1,40}$ = 10.23	**P = 0.0027 ** **
**Temperature: Hydric stress**	$F_{1,55}$ = 0.16	P = 0.6926	$F_{1,134}$ = 0.44	P = 0.5060	$F_{1,61}$ = 0.03	P = 0.8543	**$F_{1,72}$ = 24.43**	**P <0.0001 *** **	$F_{1,72}$ = 26.38	**P <0.0001 *** **	$F_{1,40}$ = 6.70	**P = 0.0134 * **
**Temperature: Flower morph**	$F_{1,55}$ = 1.24	P = 0.6270	$F_{1,134}$ = 2.59	P = 0.1069	$F_{1,61}$ = 0.75	P = 0.3894	$F_{1,72}$ = 1.70	P = 0.1963	$F_{1,72}$ = 1.99	**P = 0.0285 * **	$F_{1,40}$ = 3.05	P = 0.0884
**Hydric stress: Flower morph**	$F_{1,55}$ = 0.21	P = 0.6478	$F_{1,134}$ = 1.15	P = 0.2859	$F_{1,61}$ = 0.74	P = 0.3926	**$F_{1,72}$ = 5.46**	**P = 0.0223 * **	$F_{1,72}$ = 26.32	**P <0.0001 *** **	$F_{1,40}$ = 12.78	**P = 0.0009 *** **
**Temperature : Hydric stress : Flower morph**	$F_{2,55}$ = 1.64	P = 0.2036	F2,134 = 1.75	P = 0.1777	$F_{2,61}$ = 0.42	P = 0.6583	**$F_{2,72}$ = 5.26**	**P = 0.0074 ** **	$F_{2,72}$ = 4.78	**P = 0.0112 * **	$F_{2,40}$ = 1.55	P = 0.2243

Table S6: Results of statistical analyses (ANOVA3) for number of pollen grains per anther for the different flower morphs of F. *esculentum*. Each variety was subjected to two temperatures (21 °C vs. 28 °C) and water supply conditions (well-watered (WW) vs. water stressed (WS)). * = P <0.05, **= P < 0.01 and *** = P < 0.001. Grey boxes represent significant P-values.

	BGZ		Darja		La Harpe	
**Temperature**	$F_{1,32} = 4.74$	**P = 3.36*10^{-2} ***	$F_{1,154} = 20.83$	**P = 1.02*10^{-5} *****	$F_{1,32} = 9.96$	**P = 3.48*10^{-3} *****
**Hydric stress**	$F_{1,32} = 4.94$	**P = 3.36*10^{-2} ***	$F_{1,154} = 57.37$	**P = 3.16*10^{-12} *****	$F_{1,32} = 1.14$	P = 0.29
**Flower morph**	$F_{1,32} = 1.68$	P = 0.20	$F_{1,154} = 49.56$	**P = 5.96*10^{-11} *****	$F_{1,32} = 0.78$	P = 0.38
**Temperature: Hydric stress**	$F_{1,32} = 0.03$	P = 0.85	$F_{1,154} = 11.05$	**P = 1.10*10^{-3} ****	$F_{1,32} = 5.30$	**P = 0.03***
**Temperature: Flower morph**	$F_{1,32} = 3.23$	P = 0.08.	$F_{1,154} = 0.95$	P = 0.33	$F_{1,32} = 0.02$	P = 0.90
**Hydric stress: Flower morph**	$F_{1,32} = 2.33$	P = 0.14	$F_{1,154} = 0.34$	P = 0.56	$F_{1,32} = 1.33$	P = 0.25
**Temperature : Hydric stress : Flower morph**	$F_{2,32} = 0.02$	P = 0.96	$F_{2,154} = 3.49$	P = 0.06.	$F_{2,32} = 0.65$	P = 0.42

Table S7: Results of statistical analyses (ANOVA3) for the behaviour of insects visiting plants grown under two temperatures (21 °C vs. 28 °C) and water supply conditions (well-watered (WW) vs. water stressed (WS)). * = P <0.05, **= P < 0.01 and *** = P < 0.001. Grey boxes represent significant P-values.

Time per visit	Temperature	$F_{1,292}= 1.79$	P = 0.18
	Hydric stress	$F_{1,292}= 3.1$	P = 0.08 .
	Order	$F_{2,292}= 0.75$	P = 0.47
	Temperature : Hydric stress	$F_{1,210}= 0.80$	P = 0.37
	Temperature : Order	$F_{2,292}= 0.29$	P = 0.75
	Hydric stress : Order	$F_{2,292}= 0.42$	P = 0.66
	Temperature : Hydric stress : Order	$F_{2,292}= 2.75$	P = 0.07.
Time per flower visitation	Temperature	$F_{1,210}= 2.2*10^{-3}$	P = 0.96
	Hydric stress	$F_{1,210}= 1.26$	P = 0.26
	Order	$F_{1,210}= 0.74$	P = 0.48
	Temperature : Hydric stress	$F_{1,210}= 1.82$	P = 0.18
	Temperature : Order	$F_{1,210}= 2.53$	P = 0.08 .
	Hydric stress : Order	$F_{1,210}= 3.00$	P = 0.05 *
	Temperature : Hydric stress : Order	$F_{1,210}= 3.03$	P = 0.05 *
Number of flowers visited / insect	Temperature	$F_{1,210}= 2.56$	P = 0.11
	Hydric stress	$F_{1,210}= 2.26$	P = 0.13
	Order	$F_{1,210}= 2.10$	P = 0.12
	Temperature : Hydric stress	$F_{1,210}= 0.05$	P = 0.83
	Temperature : Order	$F_{1,210}= 0.89$	P = 0.41
	Hydric stress : Order	$F_{1,210}= 3.58$	P = 0.03 *
	Temperature : Hydric stress : Order	$F_{1,210}= 1.73$	P = 0.18
Number of flowers visited / inflorescences	Temperature	$F_{1,210}= 0.12$	P = 0.73
	Hydric stress	$F_{1,210}= 5.37$	P = 0.02 *
	Order	$F_{1,210}= 12.78$	$P = 4.79*10^{-6}$ ***
	Temperature : Hydric stress	$F_{1,210}= 0.17$	P = 0.68
	Temperature : Order	$F_{1,210}= 1.10$	P = 0.33
	Hydric stress : Order	$F_{1,210}= 6.86$	$P = 1.23*10^{-3}$ **
	Temperature : Hydric stress : Order	$F_{1,210}= 0.78$	P = 0.46

Table S8: Results of statistical analyses (ANOVA2) of the behaviour of Diptera, Hymenoptera and other insects visiting plants grown under two temperatures (21 °C vs. 28 °C) and water supply conditions (well-watered (WW) vs. water stressed (WS)). Probability values are represented through four symbols: ns = P >= 0.05, * = P <0.05, **= P < 0.01 and *** = P < 0.001. Grey boxes represent significant P-values.

		Diptera		Hymenoptera		Other	
Time per visit	Temperature	$F_{1,210}$= 2.66	P = 0.1	$F_{1,63}$ = $5*10^{-4}$	P = 0.98	$F_{1,18}$ = 0.52	P = 0.48
	Hydric stress	$F_{1,210}$= 1.48	P = 0.22	$F_{1,63}$ = 0.69	P = 0.41	$F_{1,18}$ = 1.00	P = 0.33
	Temperature : Hydric stress	$F_{1,210}$= 0.23	P = 0.63	$F_{1,63}$ = 5.80	P = 0.02 *	$F_{1,18}$ = 1.01	P = 0.33
Time per flower visitation	Temperature	$F_{1,210}$= 0.53	P = 0.46	$F_{1,63}$ =13.11	P = $5.87*10^{-4}$ ***	$F_{1,18}$ = 0.14	P = 0.70
	Hydric stress	$F_{1,210}$= 3.41	P = 0.07 .	$F_{1,63}$ = 6.14	P = 0.02*	$F_{1,18}$ = 0.76	P = 0.39
	Temperature : Hydric stress	$F_{1,210}$= 0.11	P = 0.74	$F_{1,63}$ = 23.79	P = $7.63*10^{-6}$ ***	$F_{1,18}$ = 0.29	P = 0.59
Number of flowers visited / insect	Temperature	$F_{1,210}$= 2.27	P = 0.13	$F_{1,63}$ = 2.17	P = 0.14	$F_{1,18}$ = $1.80*10^{-3}$	P = 0.97
	Hydric stress	$F_{1,210}$= 0.04	P = 0.84	$F_{1,63}$ = 4.63	P = 0.04 *	$F_{1,18}$ = 0.66	P = 0.43
	Temperature : Hydric stress	$F_{1,210}$= 0.22	P = 0.64	$F_{1,63}$ = 0.01	P = 0.51	$F_{1,18}$ = 1.64	P = 0.22
Number of flowers visited / inflorescence	Temperature	$F_{1,210}$= 3.51	P = 0.06 .	$F_{1,63}$ = 0.01	P = 0.90	$F_{1,18}$ = 0.47	P = 0.50
	Hydric stress	$F_{1,210}$= 0.88	P = 0.35	$F_{1,63}$ = 0.50	P = 0.48	$F_{1,18}$ = 1.84	P = 0.19
	Temperature : Hydric stress	$F_{1,210}$= 0.42	P = 0.51	$F_{1,63}$ = 3.86	P = 0.05*	$F_{1,18}$ = 0.08	P = 0.78

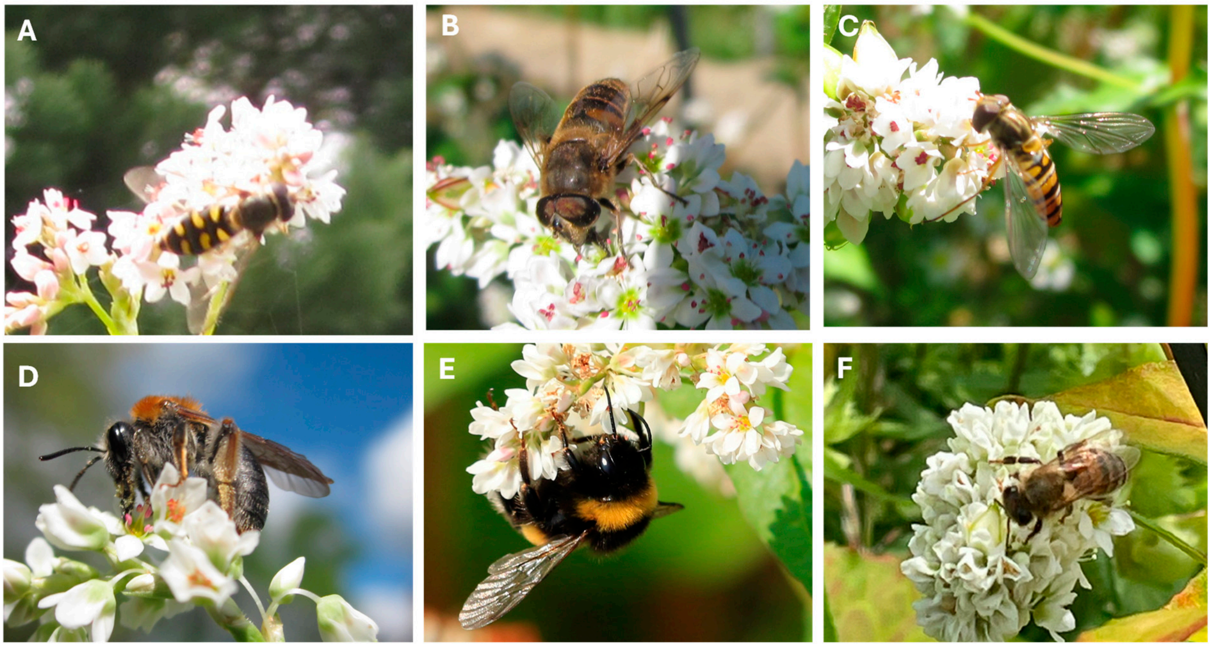

Figure S2: Some insects visiting *Fagopyrum esculentum* flowers during the experiment. (A-C) Syrphidae (Diptera), (D-F) Hymenoptera. (A) *Eupeodes* sp., (B) *Eristalis* sp., (C) *Episyrphus* sp., (D) *Adrena* sp., (E) *Bombus terrestris*, (F) *Apis mellifera*. Copyright: Jonathan Drugmand
